# Mapping the methanol-to-gasoline reaction in a fluidized bed reactor

**DOI:** 10.1039/d6ra06611a

**Published:** 2026-09-22

**Authors:** Claudia Filosa, Sohrab Askarli, Arwa Alahmadi, Juan Carlos Navarro de Miguel, Mohammad AlAbdullah, Jean Marcel R. Gallo, Javier Ruiz-Martinez, Jorge Gascon

**Affiliations:** a Advanced Catalytic Materials (ACM), KAUST Catalysis Center (KCC), King Abdullah University of Science and Technology (KAUST) Thuwal 23955 Saudi Arabia jean.gallo@kaust.edu.sa jorge.gascon@kaust.edu.sa; b Catalysis, Nanomaterials, and Spectroscopy (CNS), KAUST Catalysis Center (KCC), King Abdullah University of Science and Technology (KAUST) Thuwal 23955 Saudi Arabia; c Fuels & Chemicals Division, Research & Development Center, Saudi Aramco Dhahran 31311 Saudi Arabia

## Abstract

Spray drying was used to prepare shaped H-ZSM-5/kaolin/alumina catalysts with different silica-to-alumina ratios, and their performance was mapped in MTG under fluidized-bed conditions. Catalyst shaping preserved the zeolitic structure while decreasing surface area and modifying the acid-site distribution relative to the parent zeolites. Fixed-bed testing showed that the shaped HZ-80(S) catalyst retained comparable methanol conversion and gasoline-range selectivity to the parent H-ZSM-5, while altering the distribution of light hydrocarbons and aromatic species. Under fluidized-bed operation, the silica-to-alumina ratio strongly affected the balance between activity, stability, and gasoline-range hydrocarbon formation. For instance, HZ-23(S) deactivated rapidly, HZ-280(S) showed high dimethyl ether slip due to insufficient acidity, and HZ-80(S) provided the most favorable compromise. Mapping the effects of methanol partial pressure, temperature, and weight hourly space velocity for HZ-80(S) revealed temperature as the dominant variable controlling gasoline-range yield, with a significant temperature-space velocity interaction. A maximum gasoline-range yield of 60.7% was achieved at 400 °C and 8 h^−1^, whereas operation at 400 °C and 16 h^−1^ maintained high productivity while reducing the aromatic content of the gasoline fraction.

## Abstract 2

Spray-dried H-ZSM-5/kaolin/alumina-shaped catalysts optimize fluidized-bed methanol-to-gasoline conversion, balancing acidity, stability, gasoline yield, productivity, and aromatic control.

## Graphical abstract

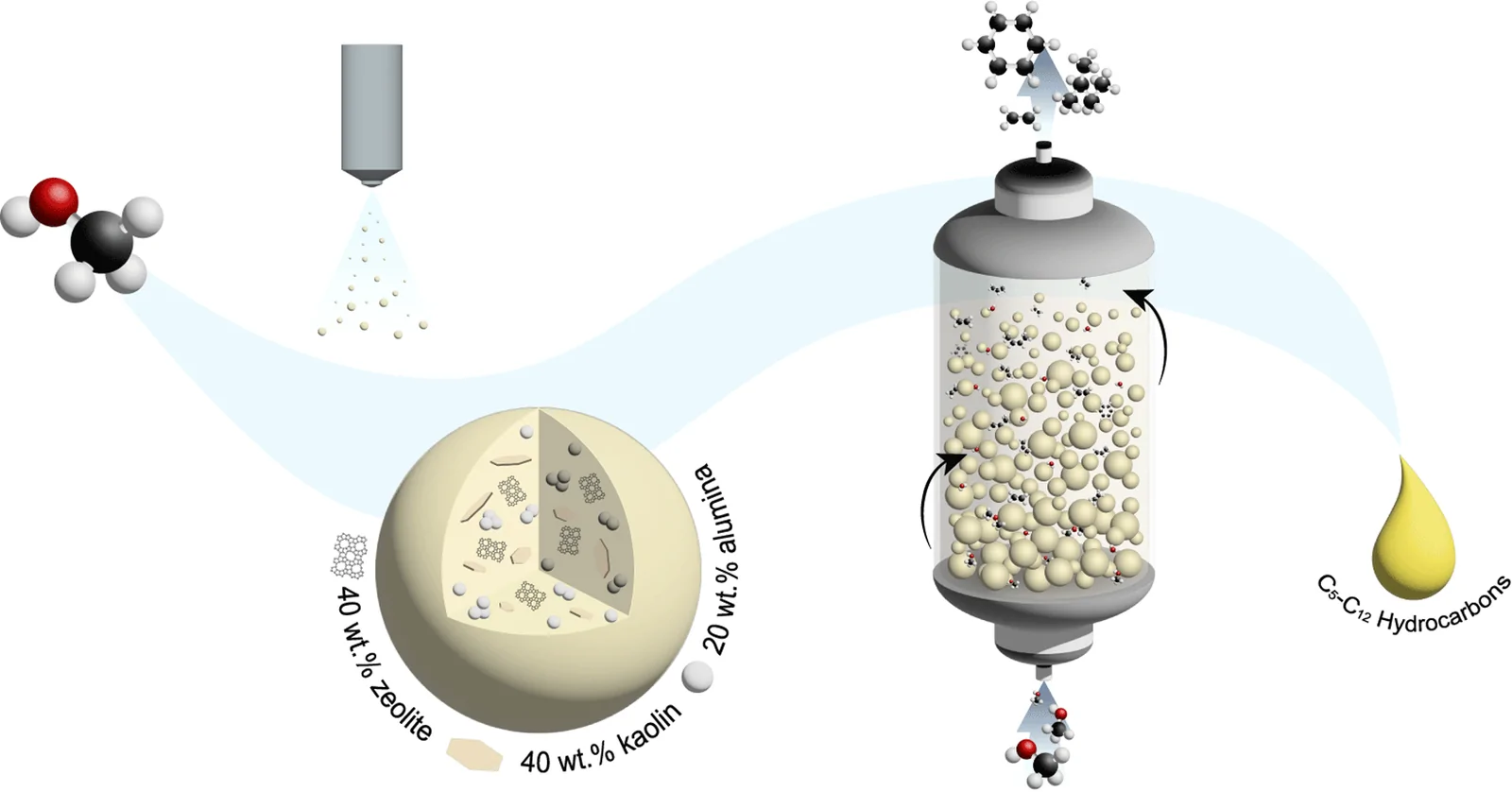

## Introduction

The growing impact of anthropogenic CO_2_ emissions on the climate has led to increasing regulatory and societal pressure on the chemical and energy industries. In this context, interest in methanol-to-gasoline (MTG) has reemerged due to its compatibility with the reaction network proposed for industrial decarbonization and net-zero emissions.

Within this framework, MTG can be carried out with e-methanol obtained by hydrogenating CO_2_ with green hydrogen.^1–3^ This process leads to CO_2_-neutral, sulphur-free fuels with an octane number comparable to gasoline.^4^

Since the discovery of MTG in the late 1970s,^5–8^ numerous studies have provided extensive overviews of the reaction.^1,9,10^ Mechanistic and parametric studies on different zeolite materials, as well as co-feeding strategies, have been extensively explored. These efforts have driven the scale-up of catalytic materials^11^ and process technologies reaching the commercial scale.^4,10^

In large-scale operations, ZSM-5 has emerged as the most extensively studied and industrially adopted catalyst for the MTG process.^4,10,12^ This zeolite acts as a selective molecular sieve and can acid-catalyze reactions due to its different types of acidity. Hydrocarbons in the boiling range of gasoline (up to C_12_) are formed in the intersecting channels of the pore structure,^12–14^ whose dimensions match those of the targeted hydrocarbons (0.56 × 0.53 nm).^13,15^ Nevertheless, catalyst deactivation and limited operational lifetime remain major challenges.^1,9^

The choice of zeolite is particularly important because MTG involves a complex network of elementary reactions, which can be divided into two stages: the induction period, during which methanol dehydration and the initial C–C bond formation occur, and the autocatalytic period, during which the conventional hydrocarbon-pool/dual-cycle mechanism dominates.^9^

Most academic and industrial research has historically focused on fixed-bed reactors.^1,9^ Alternatively, fluidized-bed MTG offers improved heat and mass transfer control, continuous catalyst regeneration, and more stable operation. These features can reduce operating costs, extend catalyst lifetime, lower carbon intensity, and improve gasoline quality.^4,10,16,17^

However, the implementation of fluidized-bed MTG depends not only on reactor design but also on the availability of catalysts with suitable physical and mechanical properties. Stable fluidization requires particles with controlled size, morphology, density, and high resistance to attrition. Accordingly, in industrial processes such as fluid catalytic cracking and methanol-to-olefins, catalysts are commonly manufactured by spray drying to produce spherical particles in the 40–100 µm size range.^18^ To achieve the required particle morphology, bulk density, and mechanical strength, the active zeolite phase is combined with a clay matrix and a binder, resulting in particles with sufficient structural integrity and low attrition.^19,20^ The resulting formulation is commonly referred to as a shaped catalyst.

Given these requirements, the choice of shaping method becomes a central issue for the development of fluidized-bed MTG catalysts. Although spray drying is the standard industrial route for producing fluidizable catalyst particles, it has received limited attention in the MTG field, where mechanical mixtures^21^ and extrusion^22–24^ have been more commonly investigated. In contrast, recent studies have demonstrated that spray-dried catalysts can be successfully applied in related methanol conversion processes operated under fluidized-bed conditions, including methanol-to-olefins^18^ and methanol-to-aromatics.^25^ These advances suggest that spray drying is a promising yet underexplored strategy for MTG catalyst design.

Building on this background, the present work evaluates spray-dried shaped catalysts based on ZSM-5 with silica-to-alumina ratios of 23, 80, and 280 in a fluidized-bed reactor. Reaction conditions were optimized to maximize gasoline-range productivity. For comparison, the same catalyst was also tested in a fixed-bed reactor to highlight the impact of reactor on product composition.

## Experimental

### Synthesis of the materials

For the methanol conversion to olefins in a fluidized-bed reactor, Gascon and co-workers^18^ have studied multiple binders and clays (fillers) and their proportions with ZSM-5. They also optimized spray-drying operational parameters. Herein, we adapted the method to prepare shaped catalysts with ZSM-5 with SiO_2_/Al_2_O_3_ ratio (SAR) of 23, 80, and 280.

First, the commercial zeolites were calcined (550 °C, 6 h, 2 °C min^−1^) to obtain the proton form. The calcined zeolite was combined with aqueous dispersions of clay and binder to obtain a composite slurry, with a final composition of 40 wt% zeolite, 40 wt% kaolin clay, and 20 wt% aluminum chlorohydrate (Al_2_Cl(OH)_5_) (Spectrum), which is the precursor for the binder. The mixture was stirred for 1 h to guarantee a homogeneous distribution of the single components.

The spray drying process to produce the shaped catalyst was performed using a lab-scale Mini Spray Dryer Buchi B-290 Advanced, operated with compressed air at 6 bar and 210 °C. A two-fluid titanium nozzle with an aperture of 2.20 mm was used to spray-dry the slurry. The liquid, continuously mixed using a magnetic stirrer, was forced through the acid-resistant nozzle using a peristaltic pump operating at a flow rate of 10 mL min^−1^, with an actual volumetric flow rate of air at standard temperature and pressure of 439 L min^−1^. The aspirator worked in suction mode at 75% of capacity. The powder from both collectors was sieved, in the range of 40–100 µm, and calcined in air in a static oven at 550 °C for 6 h, with a ramp rate of 2 °C min^−1^.

The catalysts were labelled as HZ-23, HZ-80, and HZ-280 for the plain H-ZSM-5 SAR 23, 80, and 280, while HZ-23(S), HZ-80(S), and HZ-280(S) denote their shaped counterparts.

### Catalyst characterization

Powder X-ray diffraction (PXRD) measurements were acquired in a Bruker D8 Advanced diffractometer using a Cu-Kα radiation (1.54060 Å) and operated at 40 kV and 40 mA. The data were collected in a 2*θ* angle range from 5° to 90° with a step of 0.020° and scan speed of 0.5 s per step, applying a low-angle cutting knife to avoid direct beam to the detector.

Nitrogen (N_2_) physisorption experiments were performed at −196 °C using a Micrometrics ASAP 2040 high-throughput analysis system. An initial degassing step was carried out at 350 °C under vacuum for 12 h. The specific surface areas were evaluated according to the Brunauer–Emmett–Teller (BET) method in the relative pressure range (*p*/*p*_0_) of 0.01–0.07.

Thermogravimetric analysis (TGA) was performed in nitrogen and air atmosphere using a Mettler-Toledo thermal analyser using a heating ramp of 5 °C min^−1^ in the 25–800 °C temperature range and a gas flow of 25 mL min^−1^. Three steps were performed to evaluate the total weight loss: (1) from 25 °C to 200 °C in N_2_ flow, (2) from 200 °C to 800 °C in N_2_ flow, and (3) switch to air at 800 °C.

Scanning electron microscopy (SEM) imaging was performed in a Teneo microscope using a 5 kV acceleration voltage and a 10 mm working distance. The samples were coated with a 5 nm layer of iridium prior to SEM imaging.

Temperature-programmed NH_3_ desorption (NH_3_-TPD) studies were performed in a fully automated flow Autochem 2920 system (Micromeritics Instruments Co.) with a thermal conductivity detector (TCD). For each measurement, 100 mg of material was first heated at 550 °C with a ramp of 5 °C min^−1^ under helium flow to remove the adsorbed water. Subsequently, the sample surface was saturated with ammonia at 100 °C for 2 h under a flow of helium. Finally, the desorption of ammonia was performed by heating the sample up to 600 °C (commercial zeolite) and 800 °C (shaped catalyst) under helium flow with a ramp rate of 10 °C min^−1^.

Transmission FTIR spectroscopy using pyridine as probe molecule (pyridine-FTIR) was performed in a Nicolet 6700 spectrometer with a mercury cadmium telluride (MCT-B) detector. The pellet was prepared using 15–20 mg of material and pretreated under vacuum at 450 °C for 6 hours to remove water. Pyridine vapour was then introduced at its vapour pressure under ambient conditions to saturate the pellet. The excess of pyridine was removed by evacuation at 150 °C for 1 h. Spectra were recorded in the 1000–5000 cm^−1^ wavenumber range at a resolution of 4 cm^−1^ and co-addition of 64 scans. The amount of Brønsted acid sites (BAS) were quantified from the pyridinium band near 1545 cm^−1^, while the Lewis acid sites (LAS) were obtained from the bands near 1450–1456 cm^−1^, using extinction coefficients of 1.67 and 2.22 cm·µmol^−1^, respectively.

### Catalytic testing

Catalytic tests were performed in a fluidized-bed reactor. Nitrogen was used as the carrier gas and internal standard, and fed *via* a mass flow controller (Bronkhorst). Methanol (MeOH) was fed through a syringe pump (Teledyne ISCO Syringe Pump, model 500D) in constant flow mode. The mixture was heated in an evaporator at 200 °C to ensure complete MeOH evaporation and efficient mixing with the gas phase. The N_2_/MeOH molar ratio was 1, unless otherwise specified.

The reactor, a quartz fluidized bed reactor (internal diameter 15 mm), was loaded with 4 g of catalyst. A thermocouple was inserted into the middle of the catalytic bed to maintain a constant temperature. The furnace was controlled by four thermocouples: two in control mode and the others in reading mode. Before performing the reaction, the catalyst was pretreated for 1 h under a stream of nitrogen to remove impurities at 500 °C, while the mixture of methanol and nitrogen was bypassed to collect the blank every time before the reaction.

Catalytic tests were performed at 1 bar at 325–400 °C, *P*_MeOH_ of 0.25–0.75 atm, WHSV of 4, 8 and 16 g_MeOH_ g_zeolite_^−1^ h^−1^ and shaped ZSM-5 with SAR value of 23, 80 and 280.

The product stream was analyzed using an online gas chromatograph (Agilent 7890B) equipped with a 60 m DB-1 capillary column coupled to a Flame Ionization Detector and a Thermal Conductivity Detector. Alternatively, the product stream was passed through two sequential cold traps: the first maintained at 5 °C using a recirculating chiller, and the second cooled with dry ice.

Conversion and selectivity were calculated on a molar carbon basis as described in the SI.

Liquid hydrocarbon products collected from two cold traps, separated from the aqueous layer, were analysed in a chromatographic system equipped with a flame ionization detector and fitted with a non-polar capillary column (TG-1MS, 100 m × 0.25 mm i.d., 0.50 µm film thickness, 100% dimethylpolysiloxane stationary phase) coupled with a guard precolumn to enhance resolution.

## Results and discussion

### The shaped catalyst

After calcination, the nominal composition of the shaped catalysts with different silica-to-alumina ratio (SAR) was 43.6 wt% of H-ZSM-5, 43.6 wt% kaolin clay, and 12.8 wt% aluminum oxide (aluminum chlorohydrate calcination product). The samples, sieved in the 40–100 µm fraction, exhibited spherical morphology (Fig. 1), as required for fluidized-bed applications to improve attrition resistance, minimize mass-transfer limitations, and avoid excessive pressure drop.^18–20^

**Fig. 1:**
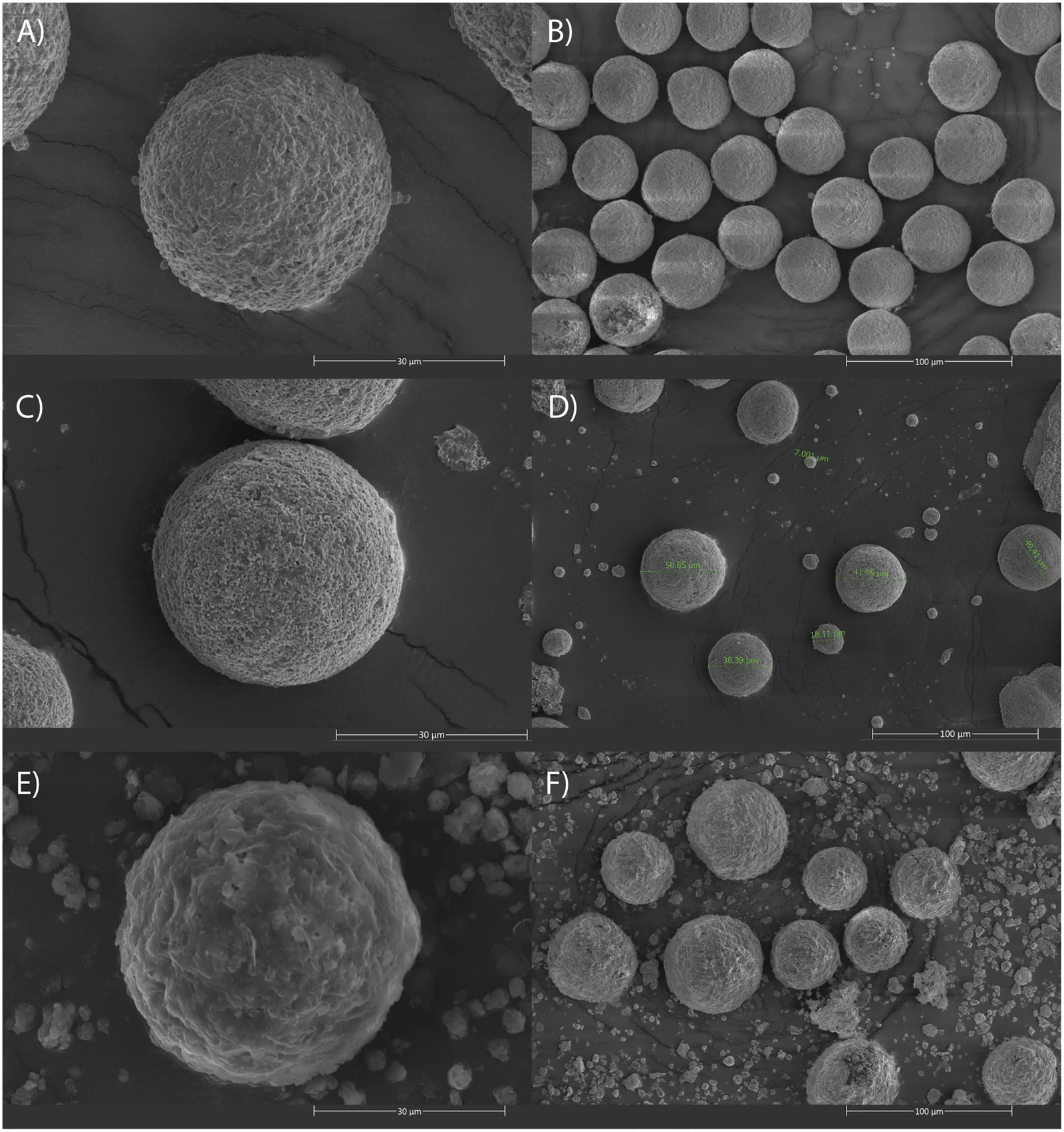
Scanning electron microscopy images of the shaped catalysts: (A and B) HZ-23(S); (C and D) HZ-80(S); (E and F) HZ-280(S). All samples were spray dried, sieved to the 40–100 µm size range, and calcined at 550 °C.

The X-ray diffraction patterns (XRD) of the shaped catalysts (41) show only reflections assigned to the zeolite phase, indicating that kaolin became largely amorphous after calcination, while the Al_2_O_3_ phase was either amorphous and/or highly dispersed. In addition, the shaped catalysts exhibited a 38–56% decrease in surface area and pore volume relative to the parent zeolites, which is expected because low-surface-area kaolin and alumina account for 56.4 wt% of the final composition (Table 1).

**Table 1:** Textural properties determined by N_2_ physisorption at −196 °C and acid sites quantification obtained by NH_3_-TPD

Material	*S*_BET_ (m^2^ g^−1^)	*V*_total_ (cm^3^ g^−1^)	Medium acidity (µmol_NH_3__ g^−1^)	Strong acidity (µmol_NH_3__ g^−1^)
HZ-23	408	0.27	696.3	484.8
HZ-80	431	0.21	344.9	439.5
HZ-280	409	0.22	169.4	281.9
HZ-23(S)	244	0.13	415.9	293.1
HZ-80(S)	225	0.13	206.9	236.2
HZ-280(S)	250	0.15	76.3	108.4

NH_3_ temperature programed desorption (NH_3_-TPD) showed that the shaped catalysts contain multiple acid-site families resulting from the combined contributions of the three components (Table 1 and Fig. S2).^26^ Desorption maxima at 170–200 °C and 325–425 °C, assigned to weak and medium acid sites, respectively, are derived from both alumina and parent zeolite phases and are commonly associated with combinations of Lewis and Brønsted acid sites.^27–30^ The concentration of these sites decreased with increasing Si/Al ratio (HZ-23 > HZ-80 > HZ-280), consistent with the lower density of framework aluminum at higher silica contents. In addition, a high-temperature peak above 500 °C was observed only in the shaped catalysts, reflecting the dehydration of kaolin and alumina.

To further distinguish the effect of shaping on the nature of the acid sites, HZ-80 and HZ-80(S) were characterized by pyridine adsorption monitored by FTIR (Fig. S3). The parent HZ-80 exhibits the characteristic bands associated with pyridine adsorbed on Brønsted (1546 cm^−1^) and Lewis (1456 cm^−1^) acid sites. These bands are also present in HZ-80(S), although with lower overall intensity, consistent with the NH_3_-TPD results. H-ZSM-5 accounts for approximately 47 wt% of the shaped catalyst, whereas the other components, alumina and kaolin, exhibit low and no measurable acidity by NH_3_-TPD, respectively. In addition, the shaped catalyst displays a new band at 1446 cm^−1^, due to pyridine coordinated to alumina Lewis acid sites. The Brønsted and Lewis acid-site concentrations (C_BAS_ and C_LAS_) were therefore quantified and normalized to the amount of H-ZSM-5 in each sample, rather than to the total catalyst mass. On this basis, the C_BAS_ of HZ-80(S) was only approximately 10% lower than that of the parent HZ-80, indicating that the intrinsic Brønsted acidity of the zeolite phase is largely retained after shaping. In contrast, the C_LAS_ increased from 52 to 92 µmol g_ZSM5_^−1^ after shaping, consistent with the additional Lewis acidity introduced by the alumina-containing matrix.

Although catalyst shaping is necessary for fluidized-bed applications, incorporating kaolin and alumina substantially alters the textural and acidic properties of the resulting catalyst, which could, in turn, affect its catalytic performance during MTG operation.

### MTG in fixed bed reactor: comparing the performance of parent and shaped ZSM-5

To assess the impact of the difference in properties of the shaped catalysts compared to the parent zeolite, ZSM-5 with a SiO_2_/Al_2_O_3_ratio of 80 (HZ-80), and the corresponding shaped catalysts HZ-80(S) were preliminarily tested for methanol-to-gasoline (MTG) reaction in a fixed-bed reactor, as shown in Fig. 2, Tables S1 and S2.

**Fig. 2:**
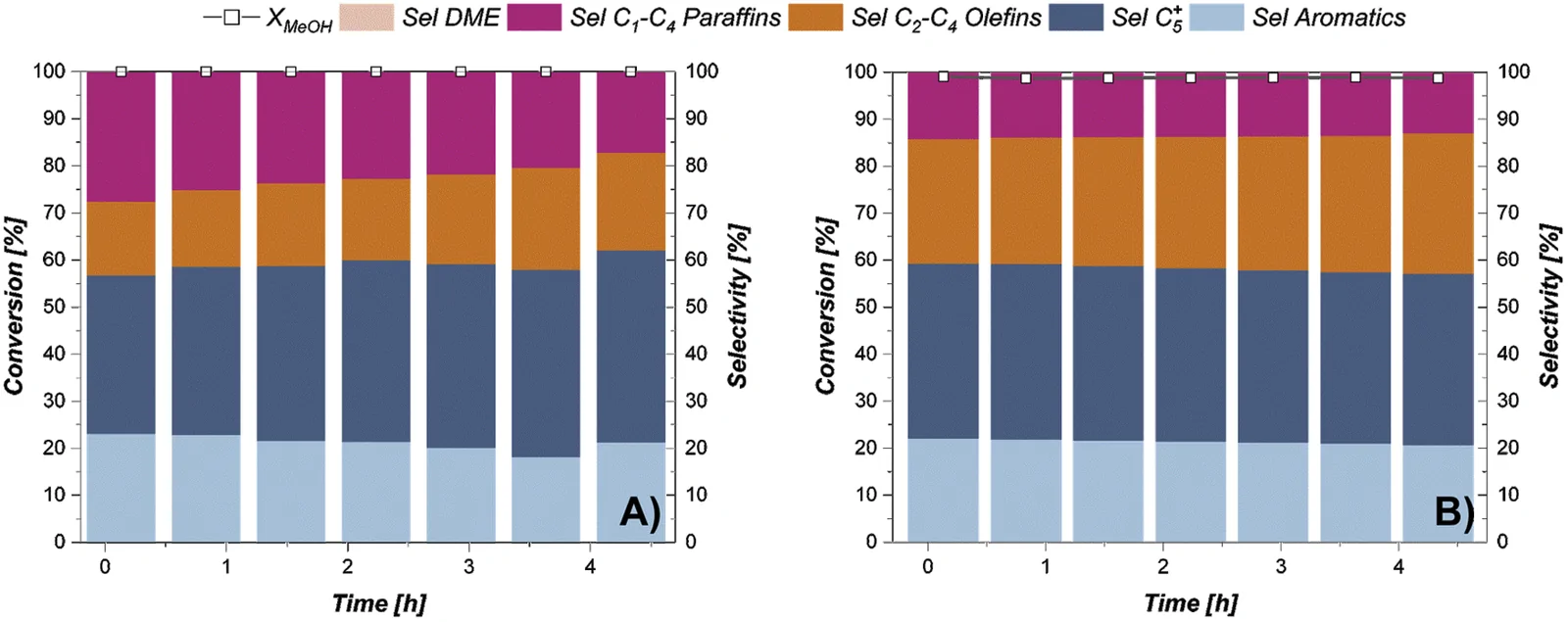
Catalytic test in fixed bed reactor of (A) the shaped catalyst HZ-80 (S) and (B) the corresponding zeolite HZ-80. Reaction conditions: *T* = 350 °C, atmospheric pressure, WHSV = 8 h^−1^, N_2_ : MeOH ratio 1 : 1.

HZ-80(S) exhibited catalytic performance comparable to that of the parent HZ-80 zeolite in terms of methanol conversion and selectivity to the gasoline-range products, *i.e.* C_5+_ hydrocarbons and aromatics. On first analysis, this suggests that the increased C_LAS_ of the shaped catalyst does not lead to a significant enhancement in cracking reactions, since the C_5+_ selectivity remains comparable, while ethylene formation is strongly suppressed.

In the first hour of reaction, however, shaping clearly altered the distribution of light hydrocarbons, most notably with a strong suppression of ethylene selectivity (2.7 *vs.* 11.7%), accompanied by higher selectivity for propane and C_4_ products. Accordingly, the ethylene-to-propylene (E/P) ratio decreased from 1.3 for the parent HZ-80 to 0.5 for HZ-80(S), further confirming that shaping modifies the distribution and subsequent transformation of light olefin intermediates.^31–33^ Previous studies on spray-dried ZSM-5 catalysts have shown that the packing and textural organization of the zeolite, clay, and binder phases can modify diffusion pathways within the catalyst particle.^18^ Such changes may alter the residence time of olefin intermediates and promote their secondary conversion prior to desorption, which could contribute to the lower E/P ratio observed for HZ-80(S).

Consistent with this trend, the olefin/paraffin ratio decreased markedly from 2.0 for the parent HZ-80 to 0.6 for HZ-80(S), indicating enhanced secondary hydrogen-transfer reactions in the shaped material (Table S1). These changes may reflect the combined influence of modified diffusion pathways^18^ and the higher C_LAS_ of HZ-80(S).^34^ This behavior suggests that, after shaping, light olefin intermediates are less likely to desorb directly as ethylene and are instead more extensively transformed into paraffins and heavier hydrocarbons.

Because aromatic selectivity remained nearly unchanged, the overall contribution of the aromatic cycle appears to remain broadly similar, indicating that the main effect of shaping was a redistribution within the olefin-mediated reaction network rather than a major shift toward aromatics. In addition, only a minor difference in Hydrogen Transfer Index (HTI) was observed (Table S1), suggesting that shaping had little effect on the overall branching character of the gasoline-range product.^31–33^ A more detailed analysis of the aromatic fraction revealed that, although the total aromatic selectivity was similar, shaping increased the BTX fraction significantly (21.0 *vs.* 14.1%), particularly toluene, xylenes, and ethylbenzene (Table S2), indicating that catalyst shaping also modifies aromatic speciation toward lighter monoaromatics. The higher Lewis acidity of HZ-80(S) may contribute to this change in aromatic speciation by promoting secondary reactions of methanol-derived and olefinic intermediates. This interpretation is consistent with previous studies suggesting that acidic binders promote the formation of oxygenated intermediates such as formaldehyde during methanol conversion, potentially contributing to BTX formation.^34^

During time-on-stream operation, HZ-80(S) also exhibited a gradual evolution in product selectivity, characterized by decreasing C_2_–C_4_ paraffin and aromatic fractions together with increasing C_2_–C_4_ olefin and C_5+_ selectivities. These trends suggest a progressive attenuation of secondary hydrogen-transfer and aromatization reactions under reaction conditions, likely due to preferential deactivation of the strongest acid sites, in line with literature observations. As a result, light olefin intermediates are less extensively converted into paraffins and aromatics and are instead redirected toward higher hydrocarbon products. Notably, no reduction in methanol conversion was observed over the evaluated period, contrasting with previous reports in MTH systems in which the presence of binders led to reduced catalyst stability.^34^

Therefore, the incorporation of kaolin and alumina modifies not only the acidic but also the diffusional environment of the catalyst, altering secondary reaction pathways while preserving overall MTG activity and liquid hydrocarbon production. Hence, the shaped catalyst HZ-80(S) maintained competitive performance while offering the particle properties required for fluidized-bed operation. These results demonstrate that the shaping procedure employed here is suitable for preserving catalytic functionality and supports the use of HZ-80(S) in the subsequent fluidized-bed reactor studies.

### MTG in fluidized bed reactor: effect of silica-to-alumina ratio in the shaped ZSM-5

To ensure proper fluidization, the minimum fluidization velocity was determined as shown in Fig. S4.^35^ Based on the calculated inlet gas velocity at the reactor entrance, the optimal operating range at 350 °C was established between 1 and 6 cm s^−1^. Accordingly, considering the reactor diameter and catalyst loading, stable fluidization could be achieved at WHSV values between 4 and 16 h^−1^.

After establishing that catalyst shaping preserves selectivity to gasoline-range products, the shaped catalysts with different SAR, HZ-23(S), HZ-80(S), and HZ-280(S) were tested in the MTG reaction under fluidized-bed conditions for 5 h on stream at 350 °C, atmospheric pressure, WHSV of 8 h^−1^, and a N_2_ : MeOH molar ratio of 1.

As shown in Fig. 3A, HZ-23(S) undergoes rapid initial deactivation, commonly attributed to coking, especially visible with the increasing availability of acid sites related to higher aluminum presence in the zeolite framework.^36,37^ Methanol conversion decreases substantially during the first hour of reaction and then stabilizes. However, DME selectivity increases along with time on stream, indicating catalyst deactivation. In contrast, HZ-80(S) and HZ-280(S) are significantly more stable, showing only minor reductions in methanol conversion and limited changes in product distribution over time on stream, due to the lower aluminum related to the Brønsted acidity (Fig. 3B and C). In addition, HZ-80(S) provides superior selectivity to gasoline-range hydrocarbons, reaching 49.1% after 3 h on stream, compared with 32.2% for HZ-280(S) and 17.1% for HZ-23(S) (Table S4).

**Fig. 3:**
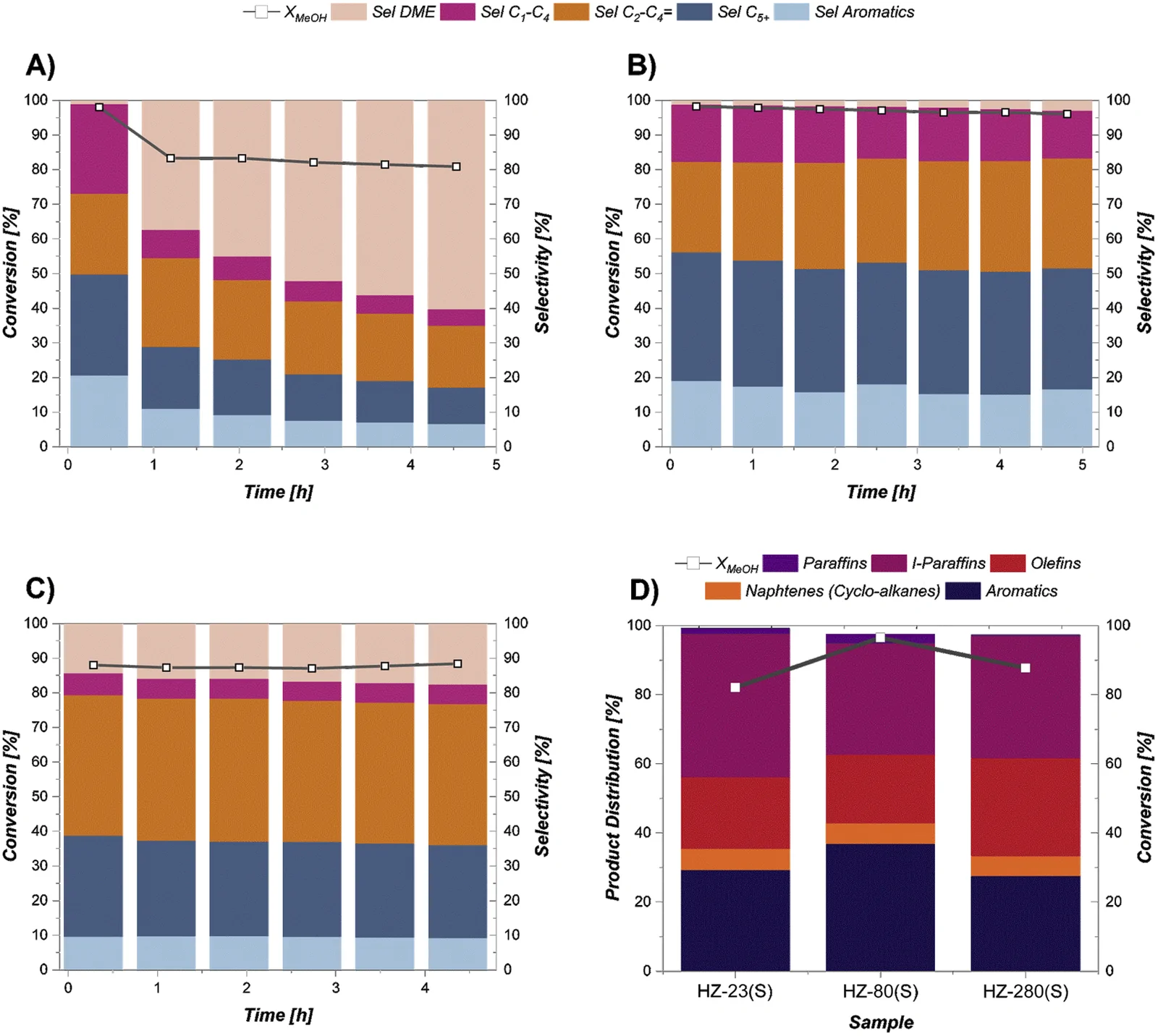
Effect of the silica-to-alumina ratio (SAR) of the shaped HZ(S) catalysts on MTG performance in a fluidized-bed reactor: (A) HZ-23(S), (B) HZ-80(S), and (C) HZ-280(S). (D) Product distribution of the condensed liquid collected after 3 h on stream. Reaction conditions: 350 °C, atmospheric pressure, WHSV = 8 h^−1^, and N_2_ : MeOH molar ratio = 1 : 1.

Compared with fixed-bed operation (Fig. 2A), HZ-80(S) in the fluidized-bed reactor (Fig. 3B) displays a more stable product distribution over time on stream, together with markedly lower selectivity to short paraffins. These differences may reflect the distinct hydrodynamic and gas–solid contacting characteristics of fluidized-bed operation.

The influence of SAR is further evidenced by both the diagnostic product ratios and the detailed C_1_–C_4_ distribution (Table S4). HZ-80(S) exhibited the lowest C_2_–C_4_ olefin/paraffin and E/P ratios, together with the highest aromatic selectivity, indicating that at full conversion this catalyst most effectively promotes the secondary transformation of light olefin intermediates into gasoline-range products,^26^ consistent with significant contributions from both the olefin and aromatic cycles. Consistent with this interpretation, HZ-80(S) showed a less olefinic and more C_1_–C_4_ paraffin product distribution than HZ-280(S). In contrast, HZ-280(S) exhibited the highest selectivity to ethylene and propylene, together with the lowest formation of methane and paraffinic products, indicating that increasing SAR, and thus decreasing aluminum, suppresses aromatization and cracking pathways and preserves lighter olefinic products. HZ-23(S) also showed significant paraffinic character, but its stronger acidity was accompanied by rapid deactivation, preventing optimal gasoline-range productivity.

The effect of SAR on product distribution can be rationalized by the role of acid sites in the consecutive reactions of the MTG network. Previous studies have demonstrated the importance of an optimal SAR for MTG reactions. At excessively low SAR, the higher density of acid sites increases the probability that light olefin intermediates undergo repeated secondary transformations before leaving the catalyst. This favours hydrogen-transfer and aromatization reactions while also increasing the formation of C_1_–C_4_ hydrocarbons, thereby diverting part of the carbon away from the gasoline range. Conversely, at excessively high SAR, the lower density of acid sites reduces the probability of these consecutive reactions, allowing a larger fraction of ethylene and propylene to remain as light olefinic products rather than being converted into heavier hydrocarbons.^18,38,39^

The analysis of the condensed liquid collected between 2 and 3 h on stream (Fig. 3D and S5) further supports the above-mentioned trends observed by online GC. In particular, HZ-280(S) yielded a more olefinic and less aromatic liquid, confirming that the higher-SAR catalyst suppresses aromatization reactions and preserves a more olefinic product distribution. In contrast, HZ-80(S) exhibited a higher aromatic contribution at the expense of olefins, consistent with its lower olefin/paraffin and E/P ratios and reinforcing the presence of a more developed secondary transformation network under fluidized-bed conditions under conditions of full methanol conversion. HZ-23(S) exhibited the highest fraction of isoparaffins, suggesting that its higher acid-site density favours skeletal isomerization and extensive secondary transformations. However, unlike HZ-80(S), this stronger acid character is also accompanied by immediate deactivation during reaction, which makes this catalyst less suitable from a stability perspective.

Overall, these results indicate that, even in the shaped form, higher SAR H-ZSM-5 tends to suppress the aromatization pathways, thereby preserving lighter olefinic products, both in gas and liquid phase. However, the best overall catalytic performance was achieved at an intermediate SAR of 80, which provides the most favourable balance between activity, stability, and gasoline-range selectivity under fluidized-bed operation.

On a more general note, a fundamental difference between fluidized and fixed bed operation lies in the degree of solid backmixing. In a fluidized bed, the vigorous gas–solid contact and continuous circulation of particles promote a more homogeneous catalyst phase, so that gas elements encounter a representative population of catalyst particles with different activity histories. In contrast, a fixed bed operates in plug flow with respect to the solid: axial gradients in reactant concentration and catalyst deactivation may develop along the bed. For reactions in which coke formation and consecutive transformations jointly determine product distribution, as is the case in methanol-to-gasoline chemistry, the local catalyst state can influence local selectivity. Consequently, solid backmixing in a fluidized bed may contribute to a more uniform activity distribution and a more stable product composition over time.

### Effect of the MTG reaction condition on the performance of HZ-80(S) in a fluidized bed reactor

The performance of HZ-80(S) was evaluated at methanol partial pressures (*P*_MeOH_) between 0.25 and 0.75, with nitrogen as the balance gas. All the experiments were performed under the same fluidization conditions. As shown in Fig. 4 and Table S4, methanol conversion decreased slightly and DME selectivity increased as *P*_MeOH_ was raised. At *P*_MeOH_ values of 0.25 and 0.50, methanol conversion remained nearly complete and relatively stable over *ca.* 5 h on stream. In contrast, at *P*_MeOH_ = 0.75, a faster decline in conversion together with increasing DME selectivity was observed, indicating the onset of catalyst deactivation.

**Fig. 4:**
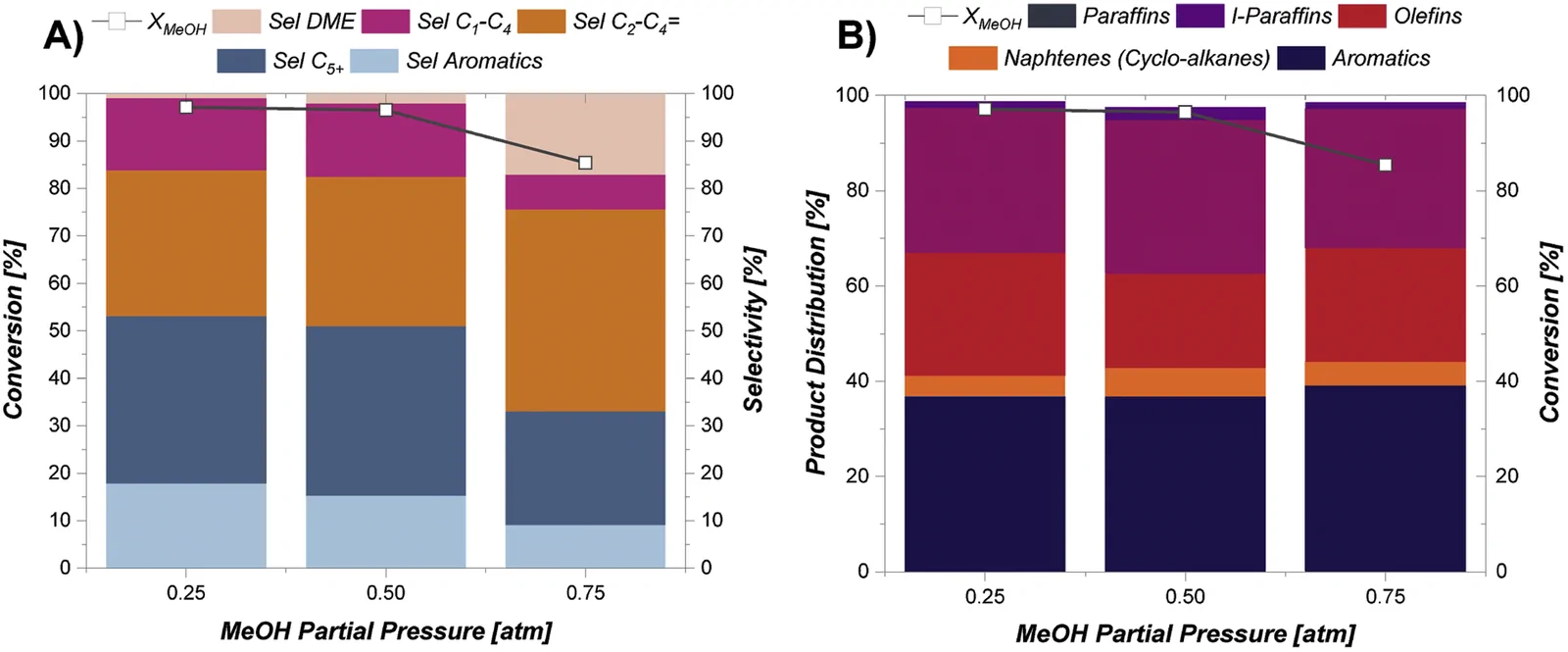
(A) Effect of different methanol partial pressure (0.25, 0.5 and 0.75) in the shaped catalyst H-ZSM-5(SC) with SAR 80. (B) Effect of different partial pressure of methanol in the liquid composition collected. Reaction conditions: *T* = 350 °C, atmospheric pressure, WHSV = 8 h^−1^, N_2_ : MeOH molar ratio 1 : 1, TOS = 3 h.

The gas-phase product distribution was similar at *P*_MeOH_ values of 0.25 and 0.50, whereas increasing *P*_MeOH_ to 0.75 markedly increased the selectivity to light olefins at the expense of the liquid fraction (C_5+_ hydrocarbons, including aromatics). The decrease catalyst lifetime with the increase of the methanol concentration is expected, as a higher methanol availability promotes the aromatic cycle and hydrogen transfer reactions,^34,40,41^ which lead to coke formation and faster deactivation. Accordingly, the gasoline-range yield decreased from 53.3% and 49.1% at *P*_MeOH_ = 0.25 and 0.50, respectively, to 28.2% at *P*_MeOH_ = 0.75 after 3 h on stream. This shift is consistent with the progressive loss of secondary transformation capacity as catalyst deactivation proceeds, reducing the conversion of primary olefin intermediates into gasoline-range hydrocarbons.

The composition of the condensed liquid collected between the second and third hour of reaction remained broadly similar under all conditions (Fig. 4B), with paraffins, olefins, and aromatics as the main product families. However, increasing methanol partial pressure slightly shifted the aromatic fraction towards more methylated species, particularly xylenes (Table S5), consistent with the higher availability of methylating intermediates under methanol-rich feeds. Thus, although operation at *P*_MeOH_ = 0.75 significantly reduced gasoline-range product yield, the overall composition of the recovered gasoline fraction is similar to that obtained with lower *P*_MeOH_. Overall, moderately diluted methanol feeds (*P*_MeOH_ = 0.25–0.50) provide the most favorable balance between catalyst stability and gasoline-range productivity.^5,42,43^

Hence, *P*_MeOH_ of 0.5 is the condition that offers the maximum feed concentration and stable operation, which led to exploring the effect of temperature (325–400 °C) and WHSV (4–16 h^−1^) on HZ-80(S) activity at this partial pressure (Fig. 5 and Table S4).

**Fig. 5:**
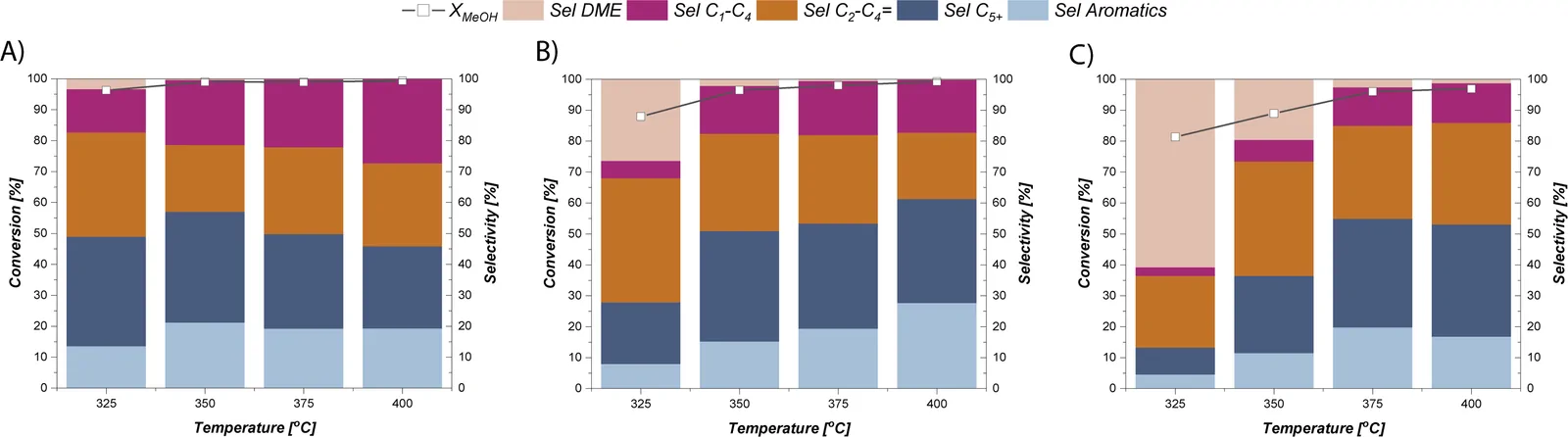
Catalytic performance of HZ-80(S) in a fluidized bed reactor at different temperatures and WHSV of (A) 4 h^−1^, (B) 8 h^−1^, and (C) 16 h^−1^. Reaction conditions: atmospheric pressure, N_2_/MeOH molar ratio = 1 : 1.

To facilitate visualization, the results were plotted as heat maps (Fig. 6). Due to the complexity of assessing the combined effect of two variables and their statistical significance, the catalytic results were evaluated using a second-order regression model that separates the effects of temperature and WHSV into linear, quadratic, and interaction terms. Given the available dataset, the model was used to describe trends and evaluate the relative effects of the operating variables within the investigated experimental range, rather than to provide general predictions beyond this domain. The corresponding model coefficients, statistical significance tests, ANOVA results, and predicted response surfaces are summarized in Tables S6–S9, Fig. S7 and S8.

**Fig. 6:**
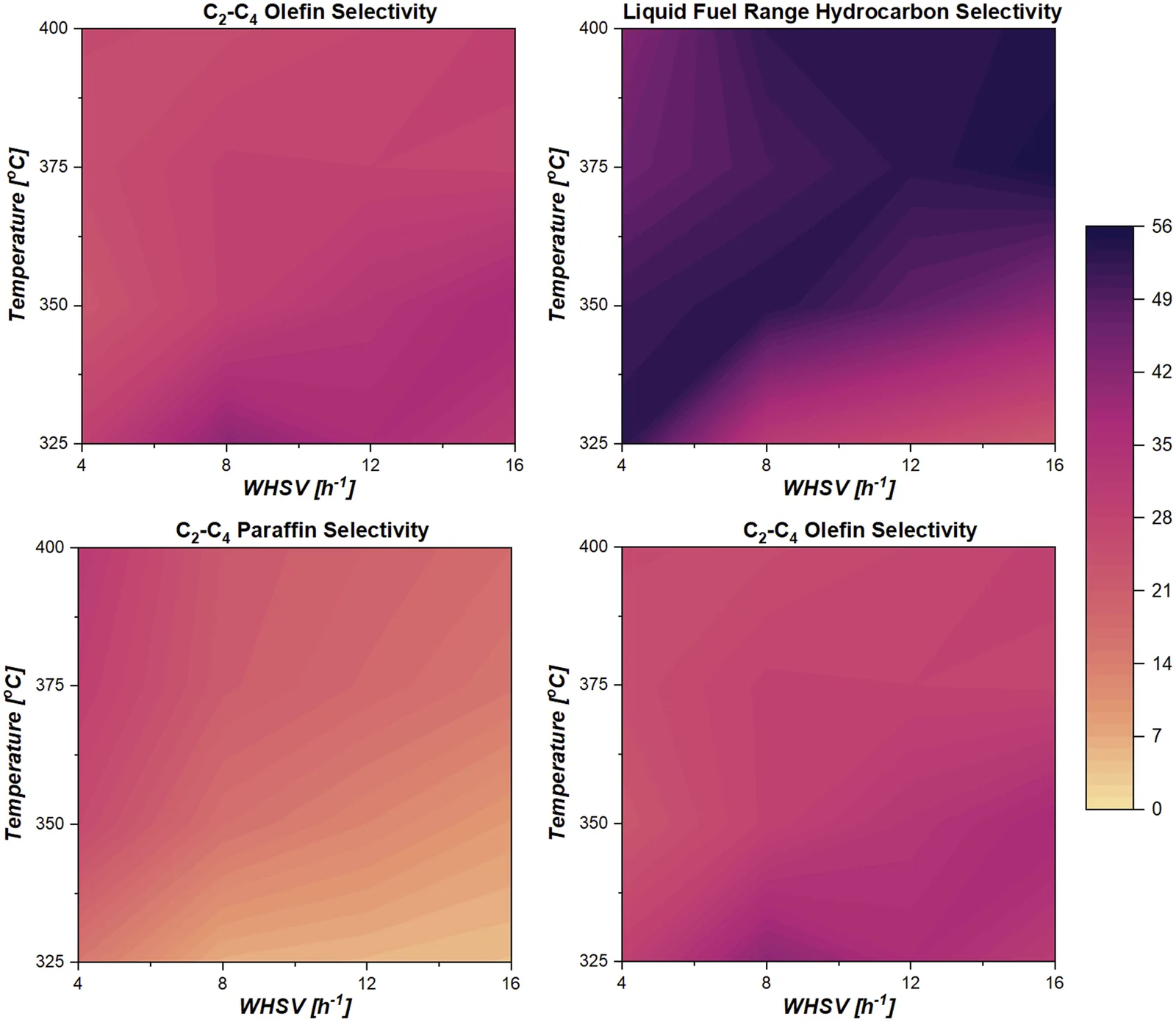
Heat map with the effect of temperature and WHSV on selectivities for different products.

The linear temperature term (*T*_c_) was positive and highly significant (*p* = 0.003), indicating that higher temperatures strongly increase gasoline-range yield. ANOVA confirmed temperature as the dominant factor, with the largest sum of squares (984.1) and the highest *F*-value (22.86). Consistent with this result, the quadratic temperature term (*T*_c2_) was negative and borderline significant, indicating that the beneficial effect of temperature is not purely linear and tends to level off near the upper end of the investigated range. ANOVA provided the same borderline significance.

For WHSV, only the linear term (*W*_c_) was statistically significant. Its negative coefficient indicates a tendency for gasoline-range yield to decrease with increasing WHSV when considered independently. However, the interaction term (*T*_c_ × *W*_c_) was positive and significant (*p* = 0.014; *F* = 11.64), demonstrating that the effect of temperature depends on WHSV. In practical terms, temperature is the primary variable controlling gasoline-range yield, whereas its positive effect becomes stronger at higher WHSV.

The second-order regression model was also used to evaluate the percentage of aromatics in the gasoline-range fraction (aromatics + C_5+_, Table S4). The linear temperature term (*T*_c_) was positive and significant (*p* = 0.010), indicating that aromatic formation increases with temperature. ANOVA confirmed temperature as the dominant factor, with the largest sum of squares (154.1) and the highest *F*-value (13.66). The interaction term (*T*_c_ × *W*_c_) was negative and significant (*p* = 0.039; *F* = 6.91), indicating that the aromatization promoted by increasing temperature becomes less pronounced at higher WHSV. Neither the quadratic temperature term nor the WHSV terms were statistically significant.

Consistent with the statistical model, the experimental data show that at both WHSV values of 8 and 16 h^−1^, the gasoline-range yield increases with temperature, reaching a maximum of 60.7% at 400 °C and 8 h^−1^ (Table S4). At the same temperature, but higher WHSV (16 h^−1^), the gasoline-range yield decreases to 51.5%; however, the aromatic content in the gasoline fraction also decreases to 31.6%, compared with 45.2% at lower space velocity. These results highlight the trade-off between maximizing gasoline-range yield and minimizing aromatic content.

Analysis of the MTG liquid products collected between the second and third hour of reaction at different temperatures and WHSV is shown in Fig. S5. The composition of the liquid products varied with both temperature and WHSV, with temperature exerting a stronger influence on product distribution. Increasing temperature generally increased the aromatic fraction while decreasing the relative contributions of paraffins, iso-paraffins, and olefins, whereas naphthenes remained a minor component under all conditions. The effect of WHSV was comparatively less pronounced, although higher WHSV tended to preserve slightly larger fractions of aliphatic products and somewhat lower aromatic contents. Overall, these results indicate that temperature is the dominant variable controlling the degree of secondary transformation in the liquid phase.

The carbon-number distributions likewise show that increasing temperature shifts the liquid composition toward the middle gasoline range, with a stronger concentration around C_8_–C_9_ and a lower contribution of heavier products. This trend is particularly evident at 375–400 °C, where C_8_ becomes the dominant carbon number under most conditions. Changes in WHSV produced more moderate effects, mainly reflected in slight broadening or narrowing of the gasoline-range distribution. The increased concentration of C_8_–C_9_ aromatic products at higher temperature is consistent with a greater extent of secondary reactions over HZ-80(S), such as olefin conversion, cyclization, hydrogen transfer, and aromatization.

Based on the fitted models, a constrained operating-window analysis was performed to identify regions within the investigated experimental range associated with high gasoline-range yield while limiting the aromatic content to a maximum of 35% (European standard EN 228). Rather than indicating an isolated maximum, the contour plot shows that the condition of 400 °C and 16 h^−1^ lies within a broader high-performance region, where gasoline-range yield remains close to the maximum while the aromatic fraction stays within the selected target window. This behavior indicates a degree of operational flexibility, since moderate variations in temperature or WHSV around the optimum are not expected to cause abrupt performance losses. In addition, the shape of the contours confirms the statistically significant temperature-WHSV interaction, indicating that WHSV's effect depends on reaction temperature.

Regarding the C_2_–C_4_ fraction, the experimental results show that increasing temperature favors paraffin formation at the expense of olefins, leading to lower olefin/paraffin ratios, which is consistent with a greater extent of secondary reactions such as hydrogen transfer and olefin conversion. In contrast, higher WHSV generally favors olefin preservation by limiting contact time and suppressing these pathways.

## Conclusions

Spray-dried shaped catalysts containing 40 wt% H-ZSM-5, 40 wt% kaolin and 20 wt% alumina and a final particle size of 40–100 µm were successfully prepared from H-ZSM-5 zeolites with silica to alumina ratios of 23, 80 and 280. The shaping procedure preserved the zeolitic phase, decreased the surface area in proportion to the inert content, and introduced an additional family of stronger acid sites associated with the kaolin–alumina matrix, while still maintaining the catalytic functionality required for MTG, as confirmed in fixed-bed experiments where HZ-80 and HZ-80(S) delivered comparable methanol conversion and gasoline-range selectivity. Under fluidized-bed conditions, the silica to alumina ratio governed both stability and selectivity: HZ-23(S) underwent rapid coke-induced deactivation, HZ-280(S) showed insufficient acid-site density and high DME slip, whereas HZ-80(S) provided the best compromise between activity, stability, and gasoline-range productivity. A statistical analysis of the operating window identified temperature as the dominant variable, with a significant temperature-WHSV interaction; the highest gasoline-range yield (60.7%) was obtained at 400 °C and 8 h^−1^, and a broad high-performance region was found around 400 °C and 16 h^−1^ that meets the EN 228 aromatic specification.

Comparison of the fixed and fluidized-bed experiments indicated differences in the evolution of product selectivity, with the fluidized-bed experiment exhibiting a more stable product distribution over time on stream. The solid backmixing and gas–solid contacting characteristics of fluidized beds may contribute to this behavior and provide advantages for catalyst management and operational stability in MTG. Hence, the present results demonstrate that spray-dried H-ZSM-5/kaolin/alumina catalysts combine the physical properties required for fluidized-bed operation with suitable catalytic performance for MTG.

## Author contributions

Claudia Filosa: methodology, investigation, validation, and writing – original draft. Sohrab Askarli: methodology, investigation, and validation. Arwa Alahmadi: methodology, investigation, and validation. Juan Carlos Navarro de Miguel: methodology, investigation, and validation. Mohammed A. Alabdullah: data curation, funding acquisition, supervision, and writing – review and editing. Jean Marcel R. Gallo: data curation, methodology, supervision, validation, and writing – original draft. Javier Ruiz-Martinez: conceptualization, data curation, funding acquisition, resources, supervision, and writing – review and editing. Jorge Gascon: conceptualization, data curation, funding acquisition, resources, supervision, writing – original draft, and writing – review and editing.

## Conflicts of interest

There are no conflicts to declare.

## Supplementary Material

RA-016-D6RA06611A-s001

## Data Availability

The data underlying this study are available in the published article and in its supplementary information (SI). Supplementary information: experimental evaluation; XRD patterns, NH_3_-TPD profiles, pyridine-FTIR profiles; fluidization curves; catalytic tests; liquid analysis based on carbon distribution; statistical analysis. See DOI: https://doi.org/10.1039/d6ra06611a.
