# Supplementary material for: Mapping the methanol-to-gasoline reaction in a fluidized bed reactor
Source: RSC Adv. 2026 Sep 22. Online ahead of print. doi: 10.1039/d6ra06611a (PMC13595584; doi:10.1039/d6ra06611a)
Supplement: Supplementary file 1 [file RA-OLF-D6RA06611A-s001.pdf]

Supplementary Information (SI) for RSC Advances.
This journal is © The Royal Society of Chemistry 2026

SUPPORTING INFORMATION

Mapping the Methanol-to-Gasoline reaction in a Fluidized Bed Reactor

Claudia Filosa[a], Sohrab Askarli[b], Arwa Alahmadi[a], Juan Carlos Navarro de Miguel[b], Mohammed A. Alabdullah,[c] Jean Marcel R. Gallo *[a], Javier Ruiz-Martinez[b] and Jorge Gascon*[a]

Table of Contents

Additional Experimental Section 3
1. Catalytic Evaluation 3
2. Statistical Analysis 3
Characterization of the material 4
1. XRD 4
2. NH_3-TPD 5
3. Pyridine-FTIR 6
Fluidization Curve for the Shaped Catalyst 7
Catalytic test 8
1. Shaped catalyst versus pristine zeolite in fixed bed reactor 8
2. Effect of SAR 8
3. Summary Catalytic Test 9
4. Liquid analysis based on carbon distribution 10
5. Statistical analysis plots 11

Additional Experimental Section

1. Catalytic Evaluation

Conversion of methanol was defined as the fraction of methanol consumed during the reaction, as follows:

$$X_{MeOH} = \frac{n_{C,MeOH}^{IN} - n_{C,MeOH}^{OUT}}{n_{C,MeOH}^{IN}} \cdot 100\ \%$$

where $n_{C,MeOH}^{IN}$ and $n_{C,MeOH}^{OUT}$ are the inlet and outlet mole of methanol. Selectivity towards the product i was calculated as follows:

$$S_i = \frac{NC_i \cdot n_i}{n_{C,MeOH}^{IN} - n_{C,MeOH}^{OUT}} \cdot 100\ \%$$

where NC_i is the number of carbons for the product i, and n_i number of moles of the product i.

The total molar flow rate at the outlet was evaluated based on the N_2 flow as an internal standard at the outlet.

To evaluate the initial carbon number and the initial moles of methanol, the mixture containing N_2 and MeOH was kept in bypass for two hours and sent to GC to evaluate the blank for each experiment.

Hydrogen transfer index was evaluated as

$$HTI = \frac{S_{i-butane} + S_{N-Butane}}{S_{C_4}}$$

2. Statistical Analysis

All statistical analyses, response-surface regressions, ANOVA calculations, and contour-map visualizations were performed in Python using the pandas, NumPy, matplotlib, and statsmodels packages.

The effects of reaction temperature and weight hourly space velocity (WHSV) on the formation of gasoline-range products (C_{5+} and aromatics) were evaluated using second-order response-surface regression. Statistical treatment was used as a descriptive tool to quantify trends within the investigated operating window and to identify favorable operating regions, rather than as a globally predictive optimization model.

Two response variables were selected for analysis. The first was the total gasoline-range product yield, given as:

$$\frac{(S_{C5+} + S_{\text{Aromatics}}) \cdot 100}{X_{\text{MeOH}}}$$

The second response variable was the percentage aromatic content in the gasoline-range fraction, defined as:

$$\frac{S_{\text{Aromatics}} \times 100}{S_{\text{Aromatics}} + S_{C5+}}$$

To improve numerical stability and facilitate interpretation of regression coefficients, the independent variables were mean-centered prior to fitting according to:

$$T_c = T - \bar{T}$$
$$W_c = W - \bar{W}$$

where T and W are the experimental values of temperature and WHSV, respectively, and $\bar{T}$ and $\bar{W}$ are their mean values in the dataset.
For each response, a second-order polynomial model including linear, quadratic, and interaction terms was fitted according to:

$$Y = \beta_0 + \beta_1 T_c + \beta_2 W_c + \beta_3 T_c^2 + \beta_4 W_c^2 + \beta_5 T_c W_c$$

where Y represents either total gasoline-range product yield or percentage aromatic content in the gasoline-range fraction, and β_i are the regression coefficients estimated by ordinary least squares.

Model quality was evaluated using the coefficient of determination (R^2), adjusted coefficient of determination (R^2_{adj}), and the overall F-test of the regression model. The statistical relevance of individual terms was assessed by analysis of variance (ANOVA). Because of the limited number of experiments and the absence of replicated runs, p-values were interpreted as indicators of relative factor importance within the explored design space rather than as strict inferential statistics. Accordingly, the models were used to support trend analysis and factor-interaction interpretation only within the investigated operating window.

Response surfaces were generated by evaluating the fitted models over a regular grid spanning the experimental domain. Temperature and WHSV were varied continuously between the minimum and maximum tested values, and the predicted responses were used to construct contour maps for total gasoline-range product yield or percentage aromatic content in the gasoline-range fraction.

To identify a practically favorable operating region, a constrained screening procedure was applied to the model predictions. A target aromatic-content window of 28–35% in the liquid fraction was selected, consistent with the experimentally accessible range observed in the dataset. Among the grid points satisfying this composition criterion, the condition providing the highest predicted liquid yield was identified as the best compromise between liquid productivity and controlled aromatization.

Characterization of the material

1. XRD

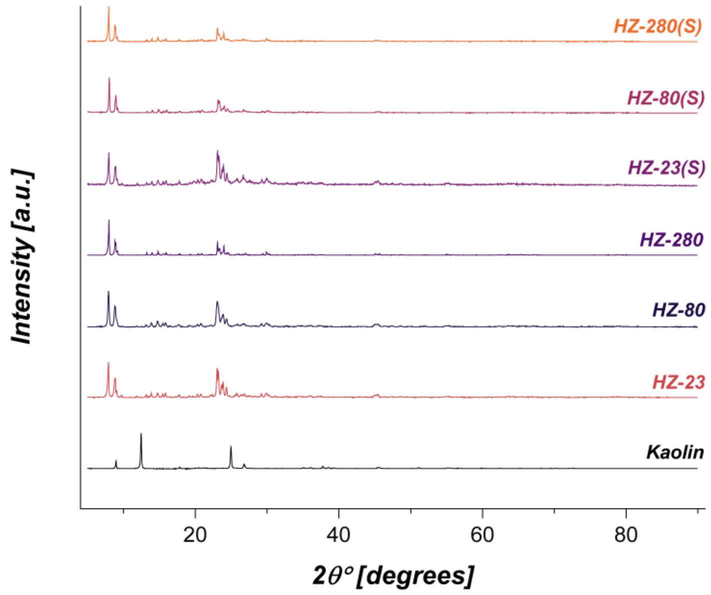

Figure S1. *Comparison of powder X-Ray diffraction patterns of kaolin clay, H-ZSM-5 SAR 23, 80 and 280 and the corresponding shaped catalyst after calcination at 550 °C.*

2. NH_3-TPD

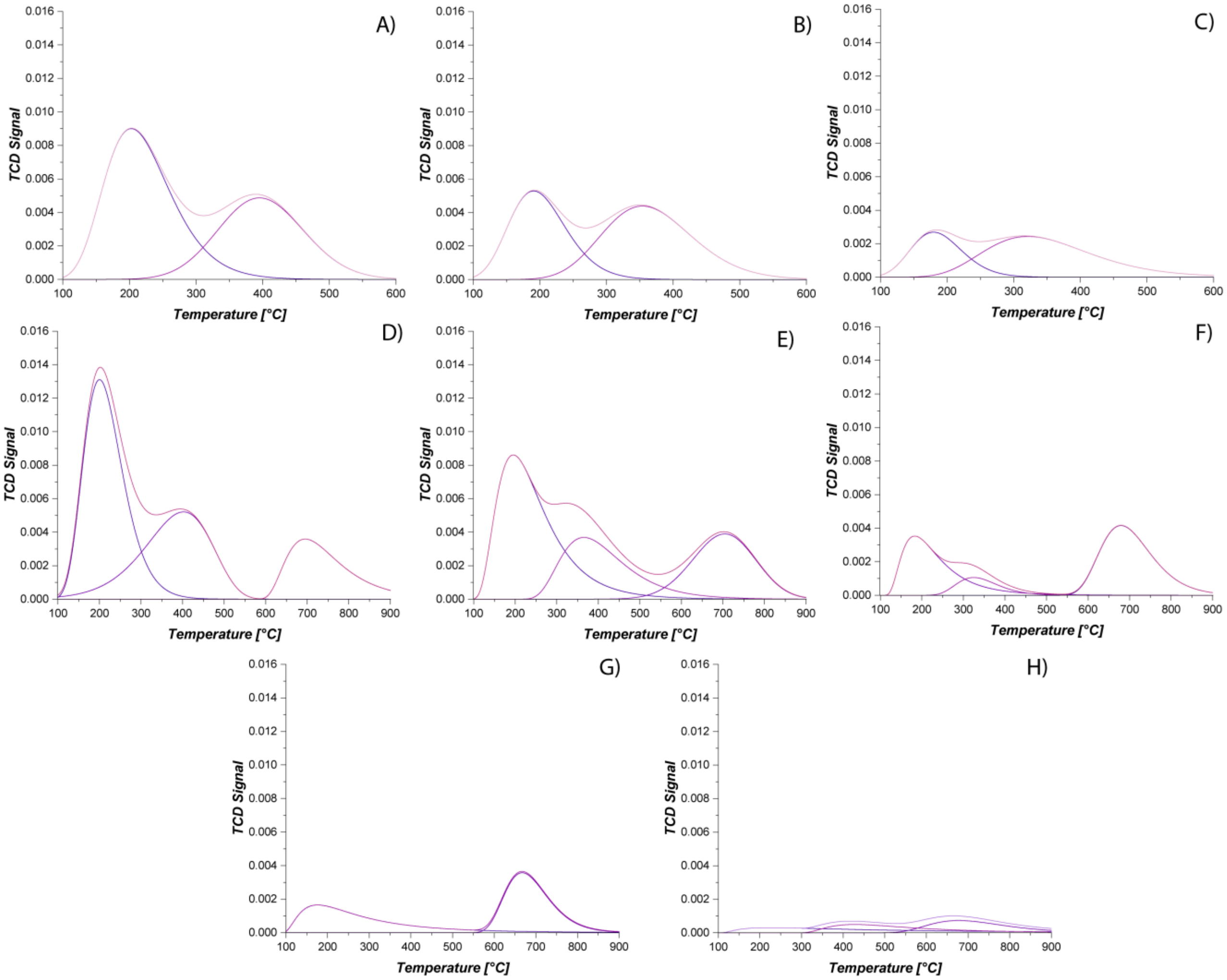

Figure S2. *NH_3-TPD profiles of the shaped catalysts sample (top) and the corresponding pristine zeolite (bottom). In order: (A) HZ-23, (B) HZ-80, (C) HZ-280, (D) HZ-23(S), (E) HZ-80(S), (F) HZ-280(S). TPD profile of Helium of (G) kaolin, and (H) alumina.*

3. Pyridine-FTIR

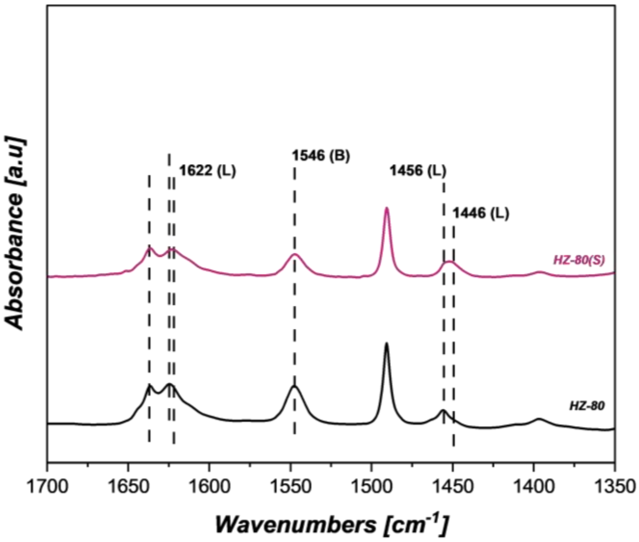

Figure S 3. *FTIR spectra of adsorbed pyridine on HZ-80 and HZ-80(S).*

Fluidization Curve for the Shaped Catalyst

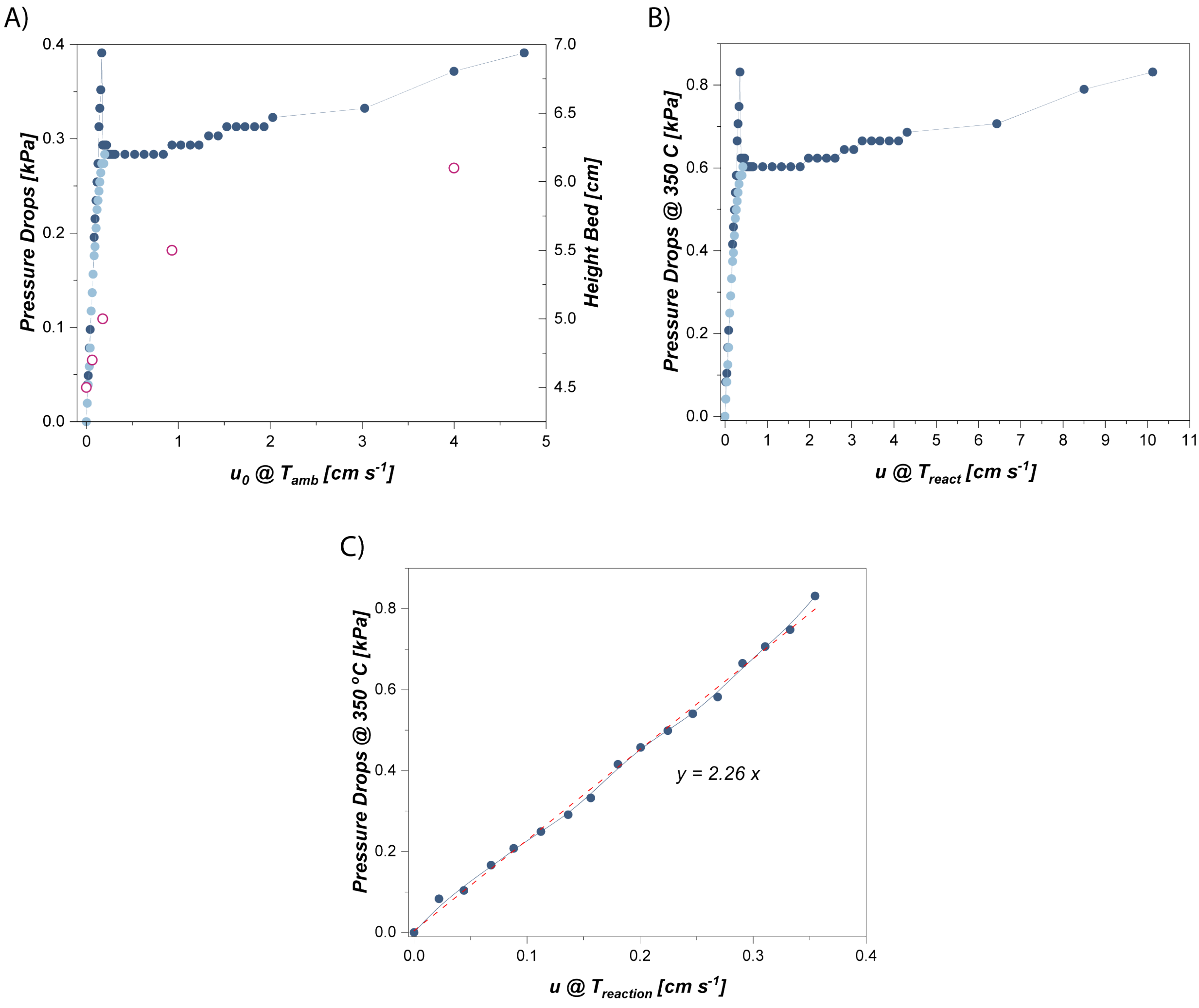

Figure S4. *Fluidization curve for the shaped catalyst HZ-80(S) at (A) ambient temperature, (B) evaluated at 350 °C and (C) linear correlation at increasing inlet velocity in the range from 0 to the minimum fluidization velocity evaluated at reaction conditions (350 °C) with the flow of nitrogen. For the curve (A) and (B), the evaluation of the minimum fluidization was evaluated considering the maximum point of the linear correlation between the pressure drops in the bed and the inlet velocity of the gas, increasing the gas velocity (in blue). The correlation between the pressure drops and the decreasing inlet velocity was obtained after crossing the minimum fluidization velocity for each case. The fluidization curve at temperature of reaction was evaluated with ideal gas law, considering the volume at the temperature of reaction (350 °C)*

Catalytic test

1. Shaped catalyst versus pristine zeolite in fixed bed reactor

Table S1. *Composition of the gas phase at 1 hour of reaction for the shaped catalyst, HZ-80(S) and the pristine zeolite HZ-80 in fixed bed mode according to the GC analysis.*

	X_{MeOH}	C_1	C_2	$C_2^=$	C_3	$C_3^=$	C_4	$C_4^=$	C_5^+	*Aromatics*	*E/P ratio*	*HTI*
HZ-80(S)	100	1.5	0.3	2.7	7.0	5.3	16.3	8.3	35.7	22.8	0.5	0.7
HZ-80	99	0.3	0	11.7	3.6	9.1	9.8	6.2	37.4	21.8	1.3	0.6

Table S2. *Detailed distribution of the aromatic fraction at 1 hour of reaction for the shaped catalyst, H-ZSM-5-(SC) (80) and the pristine zeolite H-ZSM-5(80) according to the GC analysis.*

	X_{MeOH}	*BTX*	*Benzene*	*Toluene*	*Xylenes*	*Ethyl Benzene*
HZ-80(S)	100	21	0.6	5.4	13.7	1.5
HZ-80	99	14.1	0.3	1.8	12.0	0.04

2. Effect of SAR

Table S3. *Composition of the gas phase in the first injection to GC for the three different shaped catalyst, with different SAR in the formulation. Reaction conditions: T = 350 °C, atmospheric pressure, WHSV = 8 h^{-1}, N_2 to methanol ratio 1:1.*

Sample	X_{MeOH}	*DME*	C_1	C_2	$C_2^=$	C_3	$C_3^=$	C_4	$C_4^=$	C_5^+	*Aromatics*
HZ-23(S)	98	1.1	0.5	0.8	12.5	12.1	6.3	12.5	4.5	29.2	20.5
HZ-80(S)	98	1.2	0.2	0.1	13.1	5.1	7.1	11.1	5.9	37.2	19.0
HZ-280(S)	88	14.3	0.3	0	17.2	1.8	15.4	4.3	8.0	29.1	9.6

3. Summary Catalytic Test

Table S4. *Catalytic studies of MTG in a fluidized bed reactor: Comparative performance of HZ-23(S), HZ-80(S), and HZ-280(S); and effect of the reaction conditions on HZ-80(S) performance.*

Sample	*Time [h]*	*T [ºC]*	*WHSV [h^{-1}]*	*P_{MeOH}*	*X_{MeOH} [%]*	*Selectivity [%]*									*Gasoline range Yield [%]*	*% Aromatics in Gasoline*	*E/P ratio*	*HTI*
						C_1	*C_2*	*$C_2^=$*	*C_3*	*$C_3^=$*	*C_4*	*$C_4^=$*	*C_5^+*	*Aromatics*				
HZ-23(S)	1	350	8	0.5	83	0.5	0.2	13.2	3	9.1	4.4	3.4	17.9	10.9	23.9	37.8	0.7	0.6
HZ-23(S)	3	350	8	0.5	82	0.3	0.1	10.6	2	7.8	3.5	2.7	13.4	7.5	17.1	35.9	0.7	0.6
HZ-280(S)	1	350	8	0.5	87	0.3	0	17.6	1.7	15.6	3.8	7.9	27.6	9.7	32.5	26.0	0.9	0.3
HZ-280(S)	3	350	8	0.5	87	0.3	0	17.4	1.6	15.5	3.6	7.7	27.5	9.5	32.2	25.7	0.9	0.3
HZ-80(S)	1	350	8	0.25	98.1	0.3	0.1	12.2	5.9	7.6	11.1	7.9	35.9	19.3	54.2	35.0	0.6	0.6
HZ-80(S)	3	350	8	0.25	97.5	0.3	0.2	12	6.3	7.8	11.5	6.5	34.8	19.9	53.3	36.4	0.7	0.6
HZ-80(S)	1	350	8	0.5	97.9	0.3	0.1	14.1	5	7.8	11	6.4	36.4	17.4	52.7	32.3	0.6	0.6
HZ-80(S)	3	350	8	0.5	96.5	0.3	0.1	15.5	4.5	9	10.5	7	35.7	15.2	49.1	29.9	0.6	0.6
HZ-80(S)	1	350	8	0.75	95	0.3	0.1	21.4	4.3	13.4	5.5	8.3	27.1	10.6	35.8	28.1	0.6	0.4
HZ-80(S)	3	350	8	0.75	85.4	0.3	0.1	20.9	2.9	14.3	3.9	7.3	23.9	9.1	28.2	27.6	0.7	0.3
HZ-80(S)	1	325	8	0.5	89	0.2	0.07	20.3	2.7	14.2	3.7	7.1	21.8	9.3	27.7	29.9	0.7	0.3
HZ-80(S)	3	325	8	0.5	87	0.2	0.05	19.4	2.2	14.1	3.1	6.6	19.9	8	24.3	28.7	0.7	0.3
HZ-80(S)	1	375	8	0.5	99	0.4	0.2	11.5	7.4	8.4	13.4	8.4	33.8	16.2	49.5	32.4	0.7	0.6
HZ-80(S)	3	375	8	0.5	98	0.4	0.2	11.8	5.5	8.8	11.3	8.1	34	19.4	52.3	36.3	0.7	0.6
HZ-80(S)	1	400	8	0.5	99	0.4	0.2	7.6	7	8.8	14.5	8.9	31.6	21	52.1	39.9	1.2	0.6
HZ-80(S)	3	400	8	0.5	99	0.4	0.1	6.4	5.1	7.7	11.5	7.4	33.6	27.7	60.7	45.2	1.2	0.6
HZ-80(S)	1	325	4	0.5	97	0.2	0.2	15.5	5.7	7.7	8.9	6	35.8	17.6	51.8	33.0	0.5	0.6
HZ-80(S)	3	325	4	0.5	96	0.3	0.1	18.1	5.1	9.1	8.4	6.7	35.4	13.6	47.0	27.8	0.5	0.6
HZ-80(S)	1	350	4	0.5	99	0.3	0.2	10	8.3	6.7	17	5.9	36.7	14.5	50.7	28.3	0.7	0.7
HZ-80(S)	3	350	4	0.5	99	0.3	0.2	9.5	6.3	6.4	14.1	5.7	35.8	21.2	56.4	37.2	0.7	0.7
HZ-80(S)	1	375	4	0.5	99	0.4	0.3	10	13.2	8	15.2	6.9	27.1	18.7	45.3	40.8	0.8	0.7
HZ-80(S)	3	375	4	0.5	99	0.5	0.3	10.9	8.5	9.3	12.5	7.9	30.6	19.2	49.3	38.6	0.9	0.6
HZ-80(S)	1	400	4	0.5	99	0.6	0.4	8.6	14	9.7	16.4	7.6	24.8	17.8	42.2	41.8	1.1	0.7
HZ-80(S)	3	400	4	0.5	99	0.6	0.3	8.3	11.2	10.4	15	8.1	26.5	19.3	45.3	42.1	1.2	0.6
HZ-80(S)	1	325	16	0.5	85	0.2	0	14.8	1.7	11.5	2.6	4.6	14.3	7	18.1	32.9	0.8	0.4
HZ-80(S)	3	325	16	0.5	81	0.1	0	10.8	0.9	9.3	1.7	3.1	8.7	4.6	10.8	34.6	0.9	0.3
HZ-80(S)	1	350	16	0.5	90	0.3	0.08	19.2	3	11.8	5.7	6.6	28.2	13.4	37.4	32.2	0.6	0.5
HZ-80(S)	3	350	16	0.5	89	0.3	0.05	18.3	2.2	12.5	4.4	6.1	24.9	11.5	32.4	31.6	0.7	0.4
HZ-80(S)	1	375	16	0.5	97	0.3	0.1	13.1	4.6	7.8	10.4	6.8	35.4	20.3	54.0	36.4	0.6	0.6
HZ-80(S)	3	375	16	0.5	96	0.3	0.1	14	3.3	9	8.8	7	35.1	19.8	52.7	36.1	0.6	0.6
HZ-80(S)	1	400	16	0.5	99	0.4	0.2	10.3	5.7	9.6	11.2	9.6	34.7	19.3	53.5	35.7	0.8	0.5
HZ-80(S)	3	400	16	0.5	97	0.5	0.1	12.2	3.4	11.1	8.7	9.6	36.3	16.8	51.5	31.6	0.9	0.5

Table S5. *Effect of the reaction conditions in the composition of key aromatic compounds for MTG catalysed by HZ-80(S) after 1 h of reaction.*

T* *[°C]	***WHSV* *[h⁻¹]***	P_{MeOH}	***BTX***	***Benzene***	***Toluene***	***Xylenes***	***Ethyl Benzene***
350	8	0.25	13.40	0.20	0.20	12.60	0.50
350	8	0.50	10.00	0.10	0.60	9.00	0.30
350	8	0.75	8.80	0.10	0.10	8.20	0.40
325	8	0.50	6.97	0.09	0.70	6.00	0.20
375	8	0.50	9.98	0.13	0.59	0.97	0.29
375	8	0.50	13.90	0.20	1.60	11.72	0.41
400	8	0.50	14.98	0.22	1.76	12.55	0.45
325	4	0.50	12.30	0.12	0.16	11.60	0.38
375	4	0.50	10.10	0.13	0.11	9.60	0.30
375	4	0.50	15.20	0.22	2.11	12.50	0.44
400	4	0.50	14.10	0.24	2.04	11.50	0.32
325	16	0.50	4.50	0.06	0.41	3.91	0.16
375	16	0.50	9.80	0.12	0.84	8.51	0.30
375	16	0.50	13.10	0.14	1.12	11.40	0.39
400	16	0.50	14.10	0.19	1.55	11.84	0.47

4. Liquid analysis based on carbon distribution

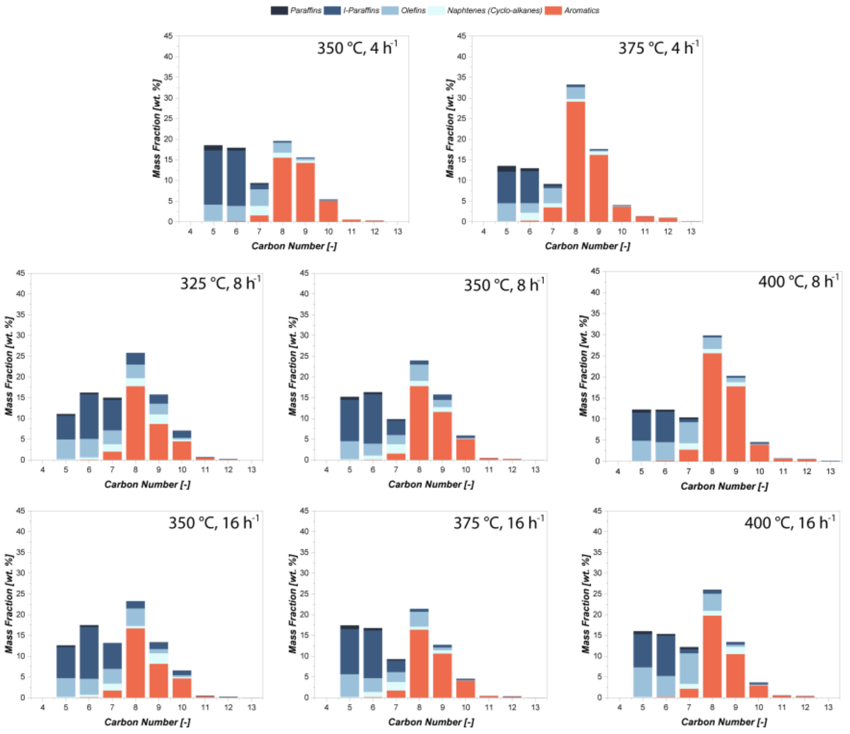

Figure S5. *Liquid distribution based on carbon number at 3 hour of reaction (liquid collected from the 2nd to the 3rd hour) for of HZ-80(S) in a fluidized bed reactor at different temperatures and WHSVs. Reaction conditions: atmospheric pressure, N2/MeOH molar ratio = 1:1.*

5. Statistical analysis plots

Heat Maps

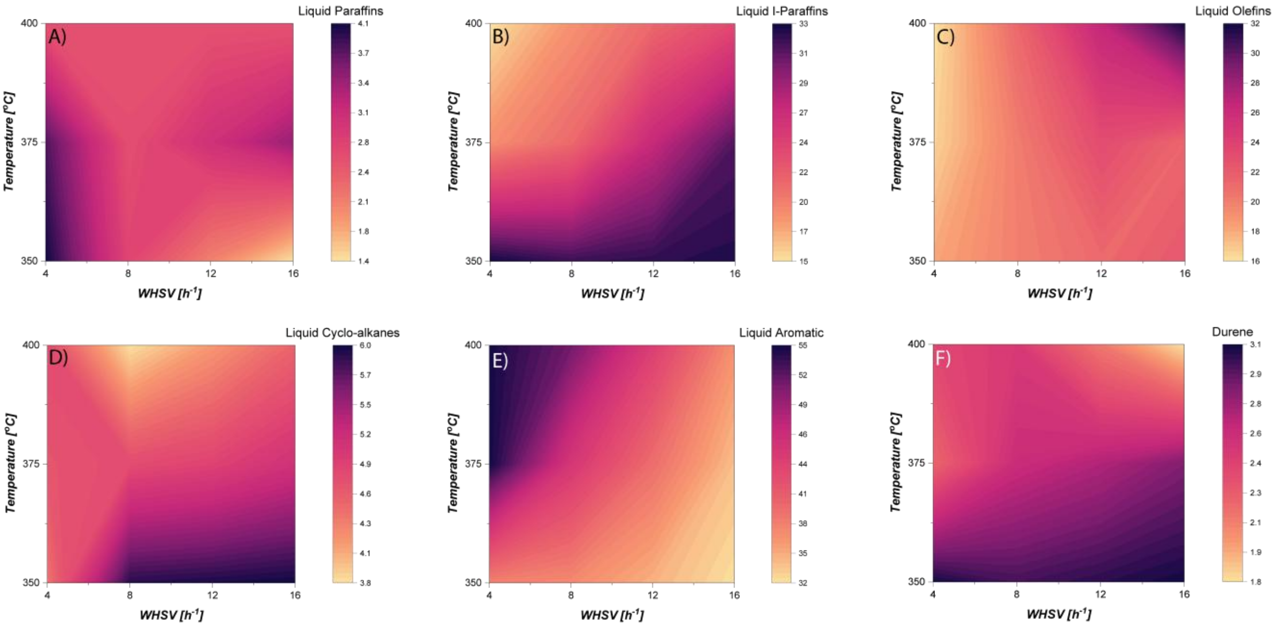

Figure S6. *Heat maps of the liquid phase based on PIONA, for the liquid collected at 3 hours of reaction: A) liquid paraffins, B) liquid i-paraffins, C) liquid olefins, D) liquid cyclo-alkanes (napthenes), E) liquid aromatic, and F) durene, based on wt.%.*

Statistical Analysis, Parameters, Analysis of Variance and Summary of Statistics

Table S6. *Summary statistics for the second-order regression models developed for gasoline-range yield and aromatic content in the gasoline fraction*

	N	R^2	Adjusted R^2	F-statistic	Model p-value
Gasoline-range yield	12	0.889	0.796	9.596	0.00791
Aromatic content in gasoline	12	0.786	0.609	4.420	0.0492

Table S7. *Regression coefficients (β), robust standard errors (HC3), t-statistics, p-values, and 95% confidence intervals for the second-order models of gasoline-range yield and aromatic content in the gasoline fraction.*

Parameter	Variable	Coefficient (β)	Robust SE (HC3)	95% CI lower	95% CI upper	p-value
**Gasoline-range Yield**	Intercept	50.7263	4.396	11.54	0	39.97
	Tc	0.324	0.068	4.781	0.003	0.158
	Wc	-1.0054	0.451	-2.229	0.067	-2.109
	I(Tc^2)	-0.007	0.003	-2.306	0.061	-0.014
	I(Wc^2)	-0.0387	0.128	-0.303	0.772	-0.352
	Tc × Wc	0.0463	0.014	3.411	0.014	0.013
**% Aromatics in gasoline**	Intercept	34.6005	2.25	15.376	0	29.094
	Tc	0.1282	0.035	3.695	0.01	0.043
	Wc	-0.2623	0.231	-1.136	0.299	-0.827
	I(Tc^2)	5.11E-05	0.002	0.033	0.975	-0.004
	I(Wc^2)	0.013	0.065	0.199	0.849	-0.147
	Tc × Wc	-0.0183	0.007	-2.629	0.039	-0.035

Table S8. *Analysis of variance (ANOVA) for the second-order regression models of gasoline-range yield and aromatic content in the gasoline fraction.*

Parameter	Source	df	Sum of Squares	Mean Square	F-value	p-value
**Gasoline-range Yield**	Tc	1	984.1338	984.1338	22.85827	0.00306
	Wc	1	347.766	347.766	8.077489	0.029486
	I(Tc²)	1	228.8657	228.8657	5.315817	0.060633
	I(Wc²)	1	3.943207	3.943207	0.091588	0.772385
	Tc × Wc	1	501.0095	501.0095	11.63684	0.014296
	Residual	6	258.3224	43.05373	—	—
**% Aromatics in gasoline**	Tc	1	154.0854	154.0854	13.65538	0.010145
	Wc	1	16.84815	16.84815	1.493119	0.267557
	I(Tc²)	1	0.012219	0.012219	0.001083	0.974816
	I(Wc²)	1	0.447758	0.447758	0.039681	0.848684
	Tc × Wc	1	77.9757	77.9757	6.910374	0.039125
	Residual	6	67.70317	11.28386	—	—

Table S9. *Summary statistics for the second-order regression models developed for gasoline-range yield and aromatic content in the gasoline fraction.*

	N	R^2	Adjusted R^2	F-statistic	Model p-value
Gasoline-range yield	12	0.889	0.796	9.596	0.00791
Aromatic content in gasoline	12	0.786	0.609	4.420	0.0492

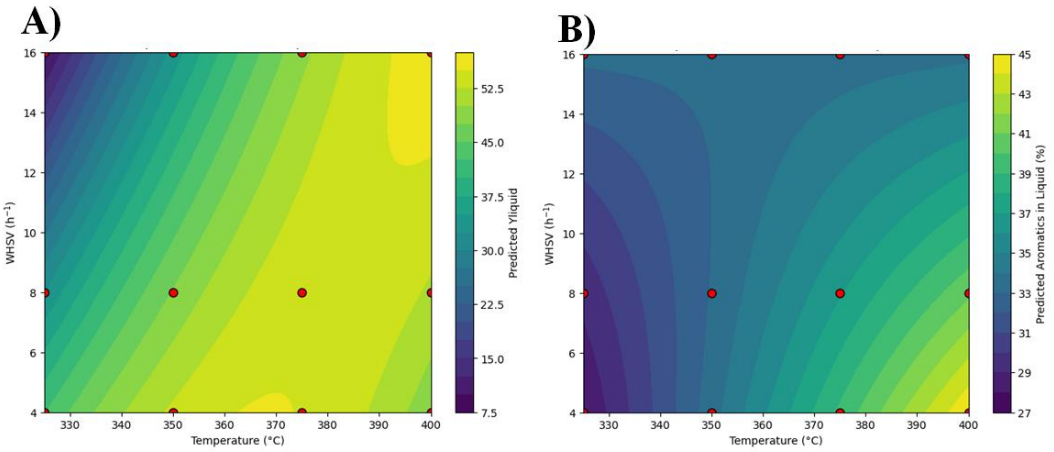

Figure S7.*Fitted response surfaces from the second-order regression models illustrating the combined effects of temperature and WHSV on (A) gasoline-range yield and (B) aromatic content in the gasoline fraction within the investigated experimental range.*

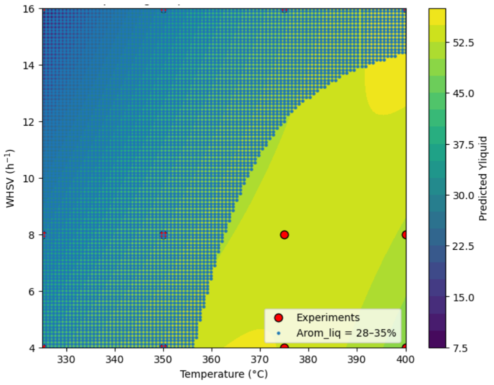

Figure S8. *Operating region identified from the fitted second-order regression models within the investigated experimental range, considering high gasoline-range yield and an aromatic content ≤35% as a function of temperature and WHSV. Red circles represent experimental runs.*
